# Spin in the Titles and Abstracts of Allergy and Immunology Randomized Controlled Trials With Nonsignificant Outcomes

**DOI:** 10.7759/cureus.84840

**Published:** 2025-05-26

**Authors:** Diana Hamdan, Abbigail Niewchas, Ambrose Loc T Ngo, Michael Weaver

**Affiliations:** 1 College of Osteopathic Medicine, Kansas City University of Medicine and Biosciences, Joplin, USA; 2 Department of Internal Medicine, Freeman Health System/Kansas City University Graduate Medical Education Consortium, Joplin, USA

**Keywords:** allergy, immunology, outcome reporting bias, randomized control trials, rct, spin

## Abstract

In scientific reporting, ‘spin’ refers to presenting neutral or negative outcomes in a manner that infers favorable results. Spin can be especially problematic in the abstracts of randomized control trials, leading to an incorrect interpretation of trial outcomes, and potentially impacting subsequent studies, patient care, and policy decisions. The present study aims to evaluate the presence of spin in the abstracts of registered randomized control trials published in allergy and immunology journals. A systematic search of the PubMed database was performed to identify randomized controlled trials of human subjects registered in a clinical trial registry with a test and control group and a nonsignificant primary endpoint. A total of 1,248 articles were screened and 66 abstracts met full inclusion criteria. Thirty-five of the 66 (53%) abstracts were found to contain one or more elements of spin, among which 11 (31.4%) had spin in the title, 29 (82.9%) in the abstract results, and 30 (85.7%) in the abstract conclusion. Industry-sponsored trials did not contain more spin compared to other funding sources (p=0.62). High rates of spin may adversely affect the interpretation and integration of new research. Careful evaluation is recommended when reviewing abstracts lacking statistically significant primary outcomes.

## Introduction and background

Randomized controlled trials (RCTs) are the cornerstone of evidence-based medicine, serving as the foundation for translating research findings into clinical practice. Despite their robustness, RCTs are often subject to distorted reporting, particularly "spin." Spin is defined as the strategic use of reporting techniques to emphasize the benefits of an experimental treatment, even when the primary outcome is statistically nonsignificant, or to distract readers from inconclusive results [1]. Common forms of spin in scientific literature include the use of misleading titles, undue emphasis on secondary or non-pre-specified endpoints, and overstating the benefits of a treatment despite a lack of statistically significant findings. Spin manipulates data presentation to align with researchers’ intended narrative, regardless of intent. This distortion can mislead readers, skew subsequent research, and influence policy development and clinical practice.

The first systematic examination of spin in 2010 revealed that over half of the reviewed RCTs with nonsignificant results contained misleading conclusions [2]. Statistically significant outcomes are preferred to studies with inconclusive or nonsignificant outcomes, which may lead to the use of spin tactics to enhance perceived impact [3]. In many such cases, superiority testing instead of non-inferiority testing, which may have been significant, could have contributed to the prevalence of spin [4]. Funding sources have also been found to play a significant role in the presence of spin [5, 6]. Studies suggest that industry-sponsored and for-profit funded RCTs are more likely than other funding sources to contain spin in their abstracts, likely caused by the vested interest of the sponsors [7, 8]. Regardless of the underlying factors driving selective reporting, addressing spin requires the rigorous review of clinical trial results and leveraging international clinical trial registries to mitigate publication bias [9].

Abstracts and titles of RCTs are particularly prone to spin due to their brevity [10]. This is especially relevant for clinicians who maintain knowledge of the latest advances in their fields by reading the abstracts of reports. As a result, medical practitioners and professionals are susceptible to improperly interpreting and applying evidence in clinical practice [11]. Similarly, lay audiences are also vulnerable to spin found in abstracts due to limited access to subscription-based journals and relying on media coverage of newsworthy studies [12, 13]. Patient health can be adversely affected by using knowledge from spin-filled literature, and numerous studies across different medical specialties have demonstrated that anywhere between 23% and 93% of abstracts and titles of published manuscripts with negative primary outcomes contained evidence of spin [14-19]. One medical field that has yet to have the prevalence of spin reported on is allergy and immunology. The primary objective of the present study was to evaluate and quantify the presence of spin in the top 10 allergy and immunology journals.

## Review

Methods

The protocol for this study was pre-published using the Open Science Framework [20].

Journal Selection Criteria

The journal selection was completed using Google Scholar Metrics h5-index as of December 2022, a measure of citation frequency over the past five years for a given journal, chosen due to accessibility and no cost to the authors. By selecting the immunology subcategory in Google Scholar metrics, the top 10 allergy and immunology journals were selected by one of the authors, M.W., in January 2023. The following journals were included: *Frontiers in Immunology*, *Immunity*, *Nature Reviews Immunology*, *Nature Immunology*, *The Journal of Allergy and Clinical Immunology*, *The Journal of Experimental Medicine*, *the Journal of Immunology*, *Science Immunology*, *Allergy*, and *Cellular and Molecular Immunology*. The search string used for article retrieval from these journals can be reviewed in Table 1.

**Table 1:** Search string used for article retrieval.

Search String
("Frontiers in Immunology"[Journal]
OR "Immunity"[Journal]
OR "Nature Reviews Immunology"[Journal]
OR "Nature Immunology"[Journal]
OR "The Journal of allergy and clinical immunology"[Journal]
OR "The Journal of Experimental Medicine"[Journal]
OR "The Journal of Immunology"[Journal]
OR "Science Immunology"[Journal]
OR "Allergy"[Journal]
OR “Cellular and Molecular Immunology”[Journal])
AND (("2010/01/01"[Date - Publication] : "3000"[Date - Publication])))
AND ("clinical trial"[Publication Type]
OR "clinical trial, phase iii"[Publication Type]
OR "clinical trial, phase iv"[Publication Type]
OR "controlled clinical trial"[Publication Type]
OR "randomized controlled trial"[Publication Type])
NOT ("clinical trial protocol"[Publication Type]
OR "clinical trial, phase i"[Publication Type]
OR "clinical trial, phase ii"[Publication Type]
OR "clinical trial, veterinary"[Publication Type]
OR "equivalence trial"[Publication Type]
OR "guideline"[Publication Type]
OR "observational study"[Publication Type])
AND (English[Language])

Article Selection Criteria

Articles found through the search string were uploaded to Rayyan, a research collaboration platform. In the first phase, articles were independently screened for inclusion as an RCT by the authors D.H. and A.L.N. in a masked fashion based on the title and abstract, with additional use of the methods section of the full-text article if further clarification was required. Inclusion and exclusion criteria are based on the original ‘Spin’ work by Boutron [2] and pre-published before the initiation of the study [20]. For inclusion, selected studies must be RCTs of human subjects registered on an available clinical trial registry with a test and control group and have a nonsignificant primary endpoint. Only studies published in English between January 1, 2010, and December 31, 2023 were considered. Language restrictions were imposed to ensure consistency of data analysis and interpretation. Date range restrictions were made to allow time for compliance with the regulatory mandate from the Food and Drug Administration Amendments Act of 2007 that all clinical trials must be registered with a clinical trial registry before patient enrollment.

In the second phase, journals were excluded if they were cluster randomized trials, phase 1 or phase 2 trials, study of multiple groups, possessed split-body trial design, lacked a primary endpoint, or failed to meet inclusion criteria. To ensure the quality of the screening process and prevent potential bias in literature collection, reports were independently selected by two authors (D.H. and A.N.). M.W., A.N., and D.H. discussed studies that did not meet either exclusion or inclusion criteria to reach a consensus.

Data Analysis

Articles meeting the inclusion criteria were independently screened for the presence of spin by two authors (D.H. and A.N.) in a masked fashion. Cohen’s kappa was used as a measure of inter-rater reliability for the assessment of spin. M.W., A.N., and D.H. discussed conflicting appraisals of spin until a consensus was reached. Data from included articles was analyzed to quantify the proportion of articles containing spin based on the journal of publication, location and type of spin, funding source, and first author’s country of origin. Funding sources were divided into one of four categories: Hospital, Private, Public, and Industry.

Studies were considered hospital-funded if the sponsor, as indicated on the clinical trial registry, was identified as a hospital or hospital network through an online search. Studies were designated as privately funded if the sponsor was determined to be a privately owned foundation or entity and included, but was not limited to, philanthropic foundations, non-profit organizations, venture capital firms, and private investment groups. Studies were designated as publicly funded if the sponsor was determined to be a government entity, such as the National Institutes of Health (NIH), the National Science Foundation (NSF), or any other government agency. Finally, studies were designated as industry-funded if the sponsor was determined to be an industry organization, such as a pharmaceutical or biotechnology company. Country of origin was identified by expanding the author affiliation feature of PubMed and recording the country listed in their respective affiliation. Statistical analysis was carried out using GraphPad Prism (GraphPad, Boston, MA, USA) to determine the odds ratio (OR), and Fisher's exact test was used to calculate a p-value for each association investigated. Data analysis was performed by D.H. and A.N.

Results

Article Selection

**Figure 1:**
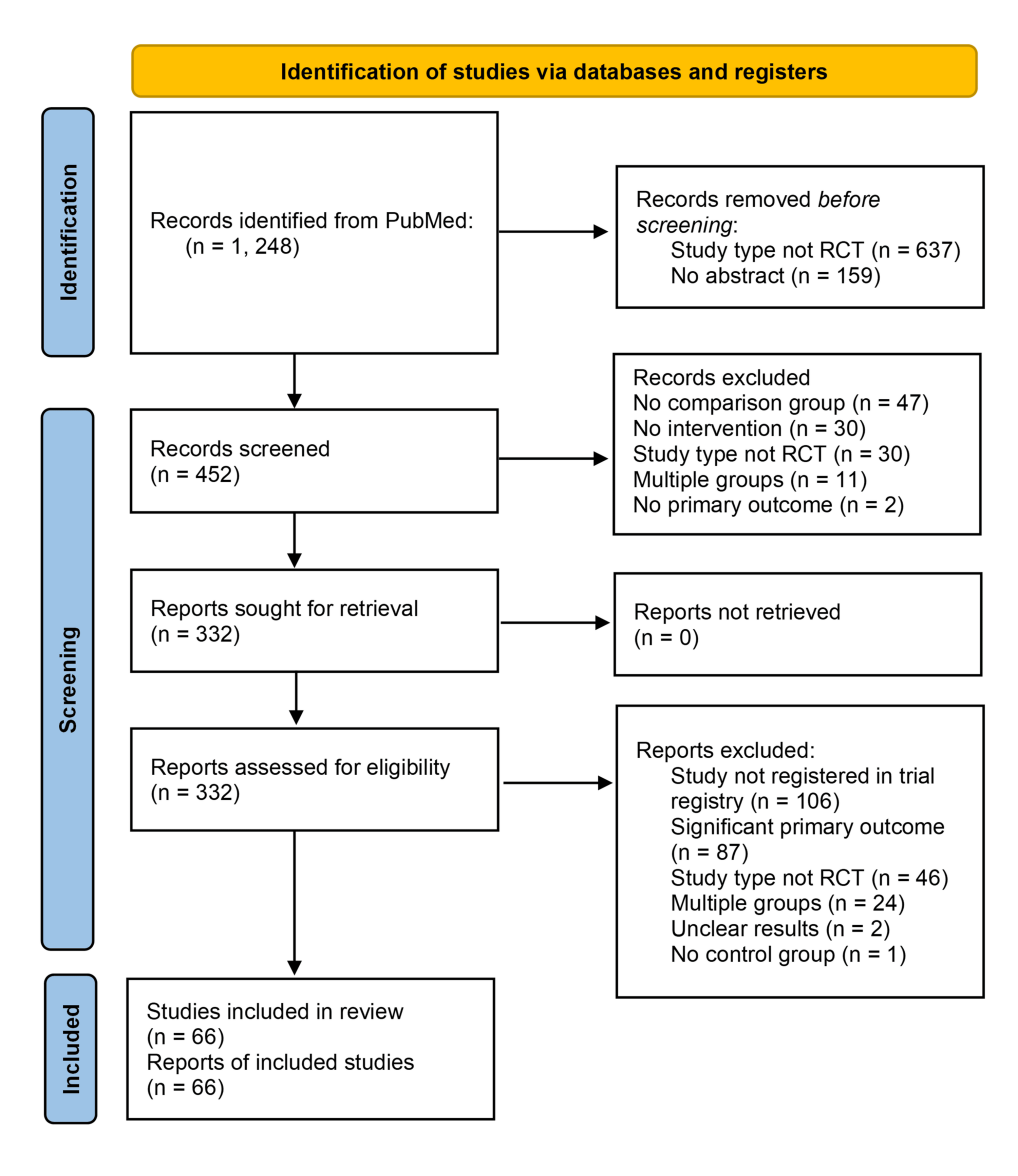
Preferred Reporting Items for Systematic reviews and Meta-Analyses (PRISMA) flow chart for the article selection process. RCT, randomized control trials

The initial search populated 1,248 articles. After the screening process, 66 articles were included for full analysis. Inter-rater reliability for the assessment of spin was substantial, with a Cohen’s kappa of 0.67. Details of the screening processes can be seen in Figure 1. Of note, *Cellular and Molecular Immunology* did not contain any relevant articles for this study.

Prevalence of Spin

Thirty-five of the 66 reports (53%) were found to contain one or more instances of spin within the title or abstract. Of the 10 journals screened, only three were found to have published reports with spin: 10 (of 12, 83.3%) articles published in *Frontiers in Immunology*, 16 (of 34, 47.1%) in the *Journal of Allergy and Clinical Immunology* (JACI), and nine (of 20, 45.0%) published in *Allergy*. Abstracts published in *Frontiers in Immunology* were over five times more likely to contain spin when compared to JACI and *Allergy* (OR 5.80, p=0.03). A summary of these findings is outlined in Table 2.

**Table 2:** Analysis of spin by journal. OR, odds ratio; CI, confidence interval * p-values were calculated using Fisher's exact test, and significance was defined as p<0.05.

Journal (2023 impact factor)	Reports with Spin, % (n/total)	OR (95% CI)	p-value*
*Frontiers in Immunology* (5.7)	83.3% (10/12)	5.80 (1.30 - 27.80)	0.03
*Journal of Allergy and Clinical Immunology* (11.4)	47.1% (16/34)	0.61 (0.23 - 1.61)	0.34
*Allergy* (12.6)	45.0% (9/20)	0.63 (0.21 to 1.73)	0.43

We further analyzed the location and type of spin in the included abstracts (Table 3). First, when analyzed by location, spin was most prevalent in the conclusion (30/35, 85.7%) and the results (29/35, 82.9%) sections compared to the title (11/35, 31.4%). Next, since multiple types of spin can be present in both the results and conclusion sections, spin frequency was calculated as a fraction of all instances observed. A total of 84 instances of spin were identified. Overall, the most common type of spin in the results and conclusion sections claimed benefit based on a statistically significant secondary endpoint (15/84, 17.9% and 16/84, 19%, respectively).

**Table 3:** Analysis of location and type of spin in sections of the abstract by journal.

		Prevalence of Spin, % (n/total)		
Spin Location	Spin Type	Journal of Allergy and Clinical Immunology	Allergy	Frontiers in Immunology
Title				
	Claiming/Insinuating benefit when none exists	13% (5/38)	14% (3/22)	13% (3/24)
Results				
	Focus on (+) secondary endpoint	16% (6/38)	9% (2/22)	29% (7/24)
	"Trend toward significance" or "Numerically larger" or similar statement	5% (2/38)	9% (2/22)	13% (3/24)
	Focus on within or between-group comparison of secondary endpoints	11% (4/38)	5% (1/22)	0
	Focus on (+) subgroup analysis	5% (2/38)	5% (1/22)	0
	Other	5% (2/38)	18% (4/22)	4% (1/24)
Conclusion				
	Claim benefit based on (+) secondary endpoint	21% (8/38)	9% (2/22)	25% (6/24)
	Claim benefit based on (+) subgroup analysis	11% (4/38)	9% (2/22)	0
	Focus on another objective (e.g., trial is (-) but they say they accomplish some goal that they did not prespecify)	8% (3/38)	5% (1/22)	0
	Claim benefit only on efficacy, ignore safety (or vice versa)	0	5% (1/22)	4% (1/24)
	Claim equivalence/non-inferiority versus control for a (-) endpoint	3% (1/38)	5% (1/22)	0
	Other	3% (1/38)	9% (2/22)	13% (3/24)

Next, we determined if the funding source influenced the likelihood that an abstract would contain at least one element of spin. As demonstrated in Table 4, no significant association was found between funding source and spin.

**Table 4:** Analysis of spin by funding source. OR, odds ratio; CI, confidence interval. * p-values were calculated using Fisher's exact test, and significance was defined as p<0.05.

Funding Source	With Spin, % (n/total)	OR (95% CI)	p-value*
Hospital	75.0% (3/4)	2.81 (0.40 - 37.6)	0.62
Private (e.g., foundation)	60.0% (3/5)	1.36 (0.26 - 8.04)	>0.99
Public (e.g., Government grant)	53.3% (16/30)	1.02 (0.38 - 2.77)	>0.99
Industry	48.1% (13/27)	0.72 (0.27 - 1.97)	0.62

When analyzed by the primary author's country of origin, the highest incidence of spin in the abstract of RCTs was identified in reports affiliated with China, Denmark, Finland, Brazil, Greece, and France. Conversely, trials conducted in Ireland, Japan, Austria, and Belgium were not found to contain any spin. A summary of these results for all countries included in this study can be found in Table 5.

**Table 5:** Analysis of spin by the primary author’s country of origin.

Country of Origin	Reports with Spin, % (n/total)
China	100.0% (5/5)
Denmark	100.0% (3/3)
Finland	100.0% (3/3)
Brazil	100.0% (2/2)
Greece	100.0% (2/2)
France	100.0% (1/1)
Germany	57.1% (4/7)
United States	46.7% (7/15)
Australia	42.9% (3/7)
Netherlands	40.0% (2/5)
United Kingdom	37.5% (3/8)
Ireland	0% (0/1)
Japan	0% (0/1)
Austria	0% (0/2)
Belgium	0% (0/4)

Discussion

The aim of the present study was to evaluate RCTs published in the top allergy immunology journals for the use of spin and explore potential factors contributing to its use. Namely, the journal of publication, funding source, and primary author country of origin were investigated as possible correlates. Of the 10 journals we screened, only articles from three journals met our inclusion criteria. Our analysis revealed an inverse relationship between the journal’s impact factor and frequency of spin. This observation lends further support to previous findings that non-inferiority RCTs published in lower-impact journals had a higher prevalence of spin in the conclusion section of the article [21]. One possible explanation for this discrepancy is variations in the rigor and extent of the peer-review process between journals. In addition, journal peer-reviewers and editors may overlook inconsistencies in reporting or even propose the introduction of spin [22]. It is worth noting, however, that reporting requirements and editorial advice were found to be insufficient to deter authors from using spin [23], calling for stronger incentives and enforcements.

Sixty-six RCTs underwent full-text cross-examination with their respective clinical trial registry to determine if the primary outcome reported in the literature matched the original intended purpose of the study. Spin was identified in 35 out of 66 RCTs (53%), most commonly in the results and conclusion sections of the abstract. This supports findings from our previous evaluation of spin in anaesthesiology RCTs, where a higher proportion of spin was found in the results and conclusion sections of the abstract compared to the title [19]. Further analysis revealed that the most common type of spin was in the form of authors claiming benefit based on a statistically significant secondary endpoint. These observations also align with previous spin studies indicating that researchers frequently emphasize secondary findings to present a more favorable presentation of their results [7, 24-26]. While journal policies surrounding the title of an article possess shared requirements of conciseness, accuracy, and informative nature, misleading interpretations of the results can still be incorporated into an abstract in a way that portrays alignment with the original purpose of a study.

When analyzed by funding source, no statistically significant relationship was identified. While this contradicts previous reports that found the industry-sponsored RCTs to contain more spin [19, 27, 28], it supports others that found no significant association [18, 29, 30]. It is plausible that research studies supported by private industries are seeing an improved transparency in data reporting and a growing emphasis on adherence to Consolidated Standards of Reporting Trials (CONSORT) guidelines [31].

Finally, analysis carried out to identify the prevalence of spin by primary author country of origin yielded interesting results. RCTs with primary authors from China, Denmark, Finland, Brazil, Greece, and France had one or more forms of spin present in all reports analyzed. In contrast, all RCTs with primary authors from Ireland, Japan, Australia, and Belgium were determined not to contain any element of spin. While regional disparities in research quality and standards may contribute to these findings [32], conclusions drawn from our results should be interpreted carefully due to the heterogeneity of the sample size of trials reviewed per country.

This study has limitations. First, there were few articles that met the inclusion criteria. Second, while more difficult to measure, it is possible that the studies we excluded based on the presence of a positive primary outcome did, in fact, contain an element of spin. Third, there is an inherent subjectivity to deeming a study as containing spin or not. Lastly, the scope of this study to include only RCTs limits the interpretation of our findings to apply to this specific study design. Other study designs may have similar rates of spin but would be difficult to investigate due to the challenge of finding pre-specified outcomes.

## Conclusions

High impact factor journals in allergy and immunology contained high rates of spin in the abstracts of RCTs with statistically nonsignificant primary endpoints. Abstracts remain a critical component of scientific research for efficient and timely review of scientific literature and the use of spin presents a threat to our ability to accurately assess data. To the best of our knowledge, this study provides the first targeted analysis into the presence of spin within the abstracts of RCTs published in allergy and immunology journals. Our findings underscore the need for researchers to adhere to transparent reporting practices based on a study's pre-specified endpoint(s). In addition, it offers scientific journals a potential approach to recognize spin and limit its use to uphold the integrity of RCT reporting. Further research could expand the scope of the inclusion criteria and number of journals to generate a more robust dataset for analysis of spin in the field of allergy and immunology.
